# The effect of multiple-enzyme treatment on *in situ* oral biofilm formation in healthy participants

**DOI:** 10.1016/j.bioflm.2025.100298

**Published:** 2025-06-21

**Authors:** Pernille Dukanovic Rikvold, Andreas Møllebjerg, Eero Juhani Raittio, Signe Maria Nielsen, Karina Kambourakis Johnsen, Marie Braad Lund, Mette Rose Jørgensen, Rikke Louise Meyer, Sebastian Schlafer

**Affiliations:** a Section for Oral Ecology, Cariology, Department of Dentistry and Oral Health, Aarhus University, Vennelyst Boulevard 9, 8000, Aarhus C, Denmark; b Novozymes A/S (part of Novonesis Group), Biologiens Vej 2, 2800, Kgs. Lyngby, Denmark; c Interdisciplinary Nanoscience Center, Aarhus University, Gustav Wieds Vej 14, 8000, Aarhus C, Denmark; d Institute of Dentistry, University of Eastern Finland, Yliopistonranta 8, 70210, Kuopio, Finland; e Section for Microbiology, Department of Biology, Aarhus University, Ny Munkegade 116, 8000, Aarhus C, Denmark

**Keywords:** Dental plaque, Enzyme Therapy, Optical coherence tomography, Confocal Microscopy, Sequence Analysis, RNA

## Abstract

Novel approaches for the prevention of biofilm-mediated oral diseases aim to control dental biofilms rather than eradicating bacteria in the mouth. One such approach is the use of enzymes that specifically target and degrade the dental biofilm matrix and thereby facilitate biofilm removal. Matrix-degrading enzymes have consistently shown promising results *in vitro*, but data on *in situ*-grown oral biofilms are limited. This study aimed to investigate the effect of combined treatment with mutanase, beta-glucanase and DNase on *in situ* biofilm formation and removal, microbial biofilm composition and biofilm pH.

Biofilms from healthy participants were grown for 48 or 72 h on lower-jaw splints and enzyme or control-treated during (3x/day, 30 min) or after growth (30 min). Under the tested conditions, enzyme treatment had no significant effect on biofilm formation or removal compared to control, as assessed by optical coherence tomography and confocal microscopy. Likewise, enzymatic treatment did not induce significant changes in the microbial composition of the biofilms that were dominated by *Streptococcus*, *Haemophilus*, *Neisseria*, *Veillonella* and *Fusobacterium* species. The biofilm pH response to a sucrose challenge was assessed using confocal microscopy-based pH ratiometry, and the average biofilm pH was not significantly different between the intervention groups. Under the conditions employed in this study, the tested enzymes had no significant impact on *in situ* grown biofilms. The treatment regimen, the biofilm composition, or the analytical methods employed may explain the difference to previous results. Further studies are warranted to assess the therapeutic potential of multi-enzyme treatment for dental biofilm control.

## Highlights

• Biofilms in an *in situ* model were treated with mutanase, beta-glucanase and DNase.
• In the model, enzymatic treatment did not remove biofilms/prevent biofilm formation.
• Biofilm pH and microbial composition in the model were not altered by the enzymes.
• The enzymes were not effective on biofilms within the limits of this *in situ* model.

## 1 Introduction

Dental caries and periodontitis, two of the most prevalent conditions of humankind, are caused by dental biofilms [1,2]. Research on these diseases therefore focuses on strategies to control dental biofilms, which often includes the use of antimicrobials [3,4]. The most widely used antimicrobial adjuncts, like chlorhexidine, quaternary ammonia compounds or essential oils, act nonspecifically on microbes, and their effect is considerably less pronounced on bacteria in biofilms than on their planktonic counterparts [[5], [6], [7]]. In part, the reduced effect is explained by the protective action of the extracellular biofilm matrix that encases and shields the bacteria, hampers the free diffusion of chemical agents and provides mechanical stability [8].

The dental biofilm matrix is rich in polysaccharides and extracellular DNA (eDNA), both of which are crucially involved in biofilm virulence [[9], [10], [11]]. Enzymes that specifically target and degrade these polymers have been proposed as an alternative means to dental biofilm control. Enzymatic treatment may destabilize the biofilms and facilitate their removal without affecting microorganisms on mucosal surfaces, and thereby contribute to the control of caries or periodontal disease without disturbing the microbial homeostasis in the mouth [12].

Studies on the effect of matrix-degrading enzymes on *in vitro*-grown biofilms consistently report favorable results. Glucanases, like mutanase and dextranase, have been shown to both prevent biofilm formation and remove established biofilms [[13], [14], [15], [16], [17]], while treatment with DNase was most effective during early stages of biofilm formation [18,19]. Most *in vitro* studies operated with simplified biofilm models consisting of only one or few species [13,16,17,20,21]. Some studies employed more complex models based on salivary inocula [15,19], but none of them match the diversity and the robustness of biofilms grown in the oral cavity in the presence of constant shear. Reports on the effect of enzymatic treatment on *in situ*-grown dental biofilms are scarce. DNase has been reported to remove oral biofilms during the first hours of growth, and to gradually lose its effect with increasing biofilm age [22]. A recent large-scale screening of multiple-enzyme combinations has identified several experimental solutions for biofilm removal, but also considerable differences between the *in vitro* and *in situ* efficacy of different enzymes [15]. It is therefore uncertain if promising results obtained for *in vitro* models can be reproduced in a clinical setting.

This study aimed to investigate a combination of the three matrix-degrading enzymes mutanase, beta-glucanase and DNase, which have recently been demonstrated to effectively prevent and remove biofilms in a saliva-based *in vitro* model. Specifically, we assessed the treatment effect on *in situ* biofilm formation and removal. Moreover, we assessed the effect on biofilm pH, as a proxy for biofilm cariogenicity, and on the microbial composition of the biofilms, as a general indicator of virulence. The null hypotheses were a) that enzyme treatment performed on established biofilms after growth does not remove the biofilms and b) that enzyme treatment performed during growth does not affect biofilm formation and biofilm pH, and change the microbial composition of the biofilms. Biofilm biovolume and thickness, as measures of biofilm accumulation, were quantified by confocal laser scanning microscopy (CLSM) and optical coherence tomography (OCT). The biofilm pH response to a sucrose challenge was quantified by CLSM-based pH ratiometry, and the microbial biofilm composition was determined by 16S rRNA gene sequencing.

## 2 Materials and methods

### 2.1 Study participants and eligibility criteria

Twelve healthy individuals were screened and enrolled in the study (mean age = 26.3 ± 7.9 SD; 9 females, 3 males). Participants had no clinical signs of active caries lesions or periodontitis, as assessed by the Nyvad criteria [23] and the periodontal screening index [24], respectively. Moreover, participants who reported to have taken antibiotic medication within 30 days prior to intervention were excluded. The study was approved by the ethical committee of Region Midtjylland (1-10-72-193-20) and conducted according to the Declaration of Helsinki and its amendments. Prior to enrolment, informed consent was obtained from all participants.

### 2.2 Enzyme solutions and product safety

The investigated enzyme solutions contained a combination of mutanase, beta-glucanase and DNase (Novozymes A/S, Kgs. Lyngby, Denmark) diluted in phosphate-citrate buffer (PCB; pH 6.0; Spectrum Chemical, New Brunswick, NJ, USA). Pure PCB, of identical appearance, served as control. Enzymes were used at a concentration of 186 μg/mL per enzyme for extraoral treatment after biofilm growth, and at 62 μg/mL for treatment during biofilm growth (see below). Prior to the study, enzymes were safety-tested both alone and in combination at 62 μg/mL for irritation of oral epithelial tissues and for genotoxicity.

### 2.3 In situ-biofilm collection

For each participant, a custom-made lower jaw splint was manufactured as described previously [25]. In brief, the splint consisted of two buccal flanges of light-cured acrylic material (Primosplint; Primotec, Bad Homburg, Germany) with 3D-printed inserts (Asiga MAX UV; Alexandria, Australia), connected by a metallic dental bar. Each insert provided space for five glass carriers (4 x 4 × 1.5 mm; 1200 grit; Menzel, Braunschweig, Germany) that were placed in a slightly recessed position (1.5 mm from the surface), which allowed for undisturbed *in situ* biofilm growth. Biofilms were grown for periods of 48 h or 72 h, during which the participants were instructed to wear the splint at all times, even during sleep, and only to remove it when eating or drinking other liquids than water or when performing regular oral hygiene procedures. To prevent dehydration of the biofilms, the splints were kept in a humid chamber whenever removed. During study intervention periods, the participants dipped the splints in 10 % (w/v) sucrose solution three times daily for 10 min (treatment during growth) or 30 min (treatment after growth).

### 2.4 Biofilm quantification

Biofilm volumes were assessed by OCT [26] and CLSM. Both methods were compared using 48-h enzyme and control-treated biofilms from three participants (biological and technical triplicates for both treatments; *n* = 54). After treatment, biofilms were fixed in 4 % paraformaldehyde for 2 h at room temperature and stored in 1:1 ethanol:phosphate buffered saline (PBS) at −20 °C.

For CLSM, microbial cells were stained with propidium iodide (PI; 15 min; 30 μM; Thermo Fischer Scientific). The glass slabs were imaged in 96-well plates for microscopy (Ibidi, GmbH, Martinsried, Germany) with a CLSM (Zeiss LSM 700, Carl Zeiss AG, Oberkochen, Germany) equipped with a 63x oil immersion objective (1.4 NA; alpha Plan-Apochromat, Carl Zeiss). PI was excited at 555 and detected from 584 to 800 nm, and the pinhole was set to 1.60 Airy Units. The acquired images had a size of 1192 x 1192 pixels (101.61 μm × 101.61 μm), a pixel dwell time of 0.68 μs, and a line average of 1. For all biofilms, three-sliced z-stacks were acquired in six predefined fields of view within the central 2 mm^2^ of the glass carriers. Images were then imported into the software daime [27], and in each image, the areas covered by bacteria were determined by intensity threshold-based segmentation. For each z-stack, total biovolumes were estimated according to the Cavalieri principle [28], as described previously [29].

For quantification by OCT, glass carriers were placed in a 96-well microtiter plate with the biofilms facing up, immersed in PBS, and scanned with a spectral domain Ganymede 620C1 OCT imaging system equipped with an LSM03 objective lens and a white light beam of 910 nm center wavelength (Thorlabs GmbH, Lübeck, Germany). The central 2 mm^2^ of the carriers were imaged with 100 kHz frequency, 83 % reference intensity and a scan averaging of 3. Images were acquired with a voxel size of 4 x 4 × 1.45 μm, resulting in 500 two-dimensional images per carrier. Biofilm thicknesses were determined using a custom-made Python script that first applied a median filter with a disk-shaped structuring element of radius 5, and then performed an intensity threshold-based segmentation (Supplementary File 1). Pixels above the threshold were counted, multiplied by the axial resolution, and averaged to calculate the mean thickness of each biofilm.

### 2.5 Enzymatic treatment after *in situ* biofilm growth

First, we assessed the effect of enzymatic treatment after accomplished biofilm growth. 72-h biofilms were collected from 11 participants (*n* = 110, 10 per participant), washed three times in PCB and immersed in PBS. Biofilm thicknesses were quantified by OCT; then biofilms from the same participant (*n* = 10) were ranked by thickness, and pairs of biofilms with similar thickness were randomized to either enzyme or control treatment using the method of randomly permuted blocks (block size of 2). Biofilm carriers were placed in 96-well plates (Nunclon Delta Surface, Thermo Fisher Scientific, Waltham, MA, USA) containing 180 μL of enzyme (186 μg/mL per enzyme) or control solution and incubated for 30 min at 37 °C with light shaking (50 rpm). Then biofilms were shaken three times in PCB for 5 s in a plate reader (17 Hz, 1020 rpm; BioTek PowerWave X52, Holm & Halby, Brøndby, Denmark). Thereafter, glass slabs were immersed in PBS and again analyzed by OCT. For quantification of the treatment effect, differences in biofilm thickness before and after treatment were compared between treatment groups.

### 2.6 Enzymatic treatment during *in situ* biofilm growth

Biofilm control may be more effective when performed during early stages of biofilm formation. We therefore also assessed the effect of enzymatic treatment during *in situ* biofilm growth. 48-h biofilms were collected from 11 participants (*n* = 220, 10 per participant and period), and treatment was performed right after the sucrose dips, 3x/day for 30 min during growth, in a two-period, split-mouth, cross-over design. The participants were blinded and allocated to a treatment sequence using random permutation. Color-coded custom-made dipping devices were used for extraoral treatment with either enzyme or control solutions. Participants received written and oral instructions on how to perform the treatment and recorded their compliance on provided sheets. For all participants, triplicate biofilms for each treatment and from each period were quantified by OCT (*n* = 132). Biofilms were fixed in 4 % paraformaldehyde for 2 h at room temperature and stored in 1:1 ethanol:PBS at −20 °C until analysis. Moreover, for each period and treatment, one biofilm from each participant (*n* = 44) was subjected to confocal microscopy-based pH ratiometry, to investigate the acidogenic potential of the biofilms. One biofilm per participant, period and treatment (*n* = 44) was analyzed by 16S rRNA gene sequencing to determine the microbial composition.

### 2.7 Ratiometric quantification of biofilm pH

Biofilms were subjected to confocal-microscopy based pH ratiometry right after collection. For period 1, the biofilm pH response to a sucrose challenge was assessed in sterile saline (pH 7.0), for period 2, the pH response was measured in cleared saliva. Paraffin-stimulated saliva samples were collected on the day of analysis from each participant, cleared by centrifugation (5 min, 1150 g) and titrated to pH 7. Sucrose was added to a concentration of 4 % (w/v) and the biofilm pH was monitored after 5 (T1), 20 (T2) and 35 min (T3; only in Period 2) at 35 °C using the ratiometric pH-sensitive probe C-SNARF-4 (30 μM; Thermo Fisher Scientific). Images were acquired at the biofilm bottom and top in nine predefined fields of view with a CLSM (Zeiss LSM 510 META; Carl Zeiss) equipped with a 63x oil objective (NA = 1.4; Plan Apochromat, Carl Zeiss). C-SNARF-4 was excited at 543 nm and detected simultaneously from 576 to 608 nm (green channel) and 629–661 nm (red channel). The pinhole was set to 1 Airy units, and the acquired images had a size of 364 x 364 pixels and a pixel dwell time of 18 μs. For each image, the average extracellular biofilm pH was determined as previously described [30]. In brief, images were exported as tif-files to the software daime [27], the red and green channel images were segmented by intensity thresholding, and all microbial cells were removed. In ImageJ [31], green channel images were divided by red channel images, and the resulting ratios (*R*) were converted to pH values according to Equation (1) [32]:

Equation 1 $$pH={\left[{\left(\frac{2.2815581}{R-0.1293069}\right)}^{\left(\frac{1}{4545673}-1\right)}\right]}^{\left(\frac{1}{8.748894}\right)}x\;34.62357$$

Calibration of C-SNARF-4 was performed using MES buffer solutions, adjusted to pH 4.0–7.8 in steps of 0.2 pH units, as described previously [33]. In brief, images were acquired of each buffer solution (50 mM) containing C-SNARF-4 (30 μM) using the same microscope and laser settings as above. *R* was calculated and plotted against the pH values, and the MyCurveFit Data Analysis Tool (My Assays Ltd., Brighton, UK) was used to fit a function to the data, resulting in Equation (1).

### 2.8 16S rRNA gene sequencing

The bacterial composition of enzyme- and control-treated biofilms was determined by next-generation 16S rRNA gene amplicon sequencing. Collected biofilms were immediately stored in extraction buffer [34] at −20 °C until analysis. DNA was extracted using an enzymatic pre-treatment [35], followed by the DNeasy PowerLyser Powersoil kit (Qiagen, Venlo, Netherlands) according to the manufacturer's protocol. The V3–V4 region of the bacterial 16S rRNA genes was amplified using primers Bac 341F and Bac 805R [36] and prepared for amplicon sequencing according to Illumina's 16S Metagenomic Sequencing Library Preparation guide. Paired-end sequencing (2 × 300 bp) was performed on an in-house Illumina MiSeq sequencer using the V3 sequencing kit (Illumina, San Diego, CA, USA).

### 2.9 16S rRNA gene sequencing analysis

All analyses were conducted in R v4.4.2 R Core Team [37]. Sequences were trimmed to remove barcodes and primers using cutadapt v0.2.0 [38]. Error correction, amplicon sequence variant (ASV) calling, chimera removal, and taxonomic classification were performed with the R package ‘DADA2’ v1.28.0 [39]. The Silva SSU reference database no. 138 [40] was used for taxonomic classification. ASVs were filtered to include only those classified as Bacteria, and nucleic acid extraction blanks and PCR negatives were used for decontaminating the data using the R package Decontam v1.20.0 [41]. Putative contaminants were identified using the prevalence method with a threshold of 0.1 and were subsequently removed from the data. Differential abundance analysis of paired samples was conducted in ANCOM-BC2 v2.2.2 [42]. Principal component analysis was performed on centered log-ratio transformed read counts. All further data analyses were conducted using the R packages Phyloseq v1.44.0 [43], Microbiome v1.22.0 [44], vegan v2.6.4 [45], ggplot2 v3.5.1 [46], and custom R scripts. All sequencing data were submitted to BioProject (accession number PRJNA1207898).

### 2.10 Statistical analysis

Gamma regression with a log link function was used to estimate the effect of enzymatic treatment applied after *in situ* biofilm growth on biofilm biovolumes determined by OCT and CLSM. For 72-h biofilms, the post-treatment thickness determined by OCT adjusted for the pre-treatment thickness in the analysis. Likewise, gamma regression with a log link function was used to estimate the effect of treatment during growth after a 48-h intervention period. Based on the joint application of multiple outlier detection algorithms (Z-scores; Interquartile range; Cook's distance; Equal-Tailed Interval), we excluded biofilm values which were classified as outliers by at least half of the methods used [47]. The effect of enzymatic treatment on biofilm pH was estimated separately for each period and time point, using linear regressions. Cluster (*i.e.* participant) bootstrapped standard errors for the final estimates were generated for all mentioned analyses. Coefficient analysis with Pearson correlation was performed to compare biovolumes quantified by CLSM and OCT.

Statistical analyses were performed using R v.4.4.1 R Core Team [37] and GraphPad Prism (v.10; GraphPad Software Inc., San Diego, CA, USA*). P* values below 0.05 were considered statistically significant.

## 3 Results

### 3.1 Effect of enzymatic treatment after *in situ* biofilm growth

**Fig. 1:**
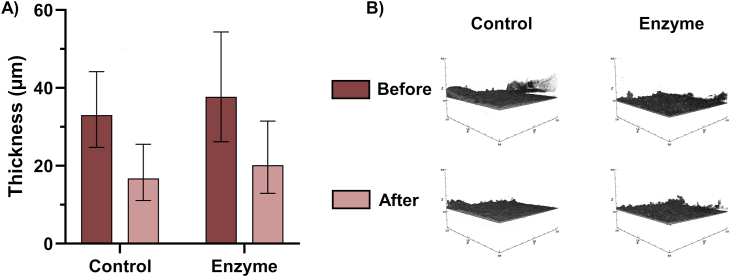
**The effect of enzymatic treatment on biofilm removal.** Biofilm thickness was measured by OCT**. A)** The biofilm thicknesses decreased after treatment with both enzyme and control solutions (*∗p* < 0.05), and there was no significant difference between the two groups (*p* = 0.73, gamma regression with log link function). Data from 95 biofilms (enzyme: *n* = 48; control: *n* = 47) from 11 subjects are shown (missing data: *n* = 12; outliers: *n* = 3). Bars and error bars show means and the upper and lower limits of 95 % confidence intervals, respectively. **B)** Representative 3D renderings of oral biofilms generated from images acquired by OCT before and after treatment.

Enzymatic treatment after biofilm growth had no significant effect on biofilm biovolumes, compared to control treatment. OCT analyses were carried out before and after treatment and showed that the washing protocol removed loosely adhering biofilm in both intervention groups (Fig. 1). The predicted response values, adjusted for pre-treatment biofilm thickness, were similar in the two groups (Enzyme: 16.0 μm, 95 % CI: 11.6–21.9 μm; Control: 15.3 μm, 95 % CI: 10.7–21.8 μm) with a ratio of means (enzyme/control) of 1.05 (95 % CI: 0.79–1.39; *p* = 0.73). Residual biovolumes after treatment were also quantified by CLSM (Table S1). No significant difference between intervention groups were found, with a ratio of means (enzyme/control) of 1.09 (95 % CI: 0.92–1.29; *p* = 0.58). Null hypothesis a) was accepted. Comparative analyses showed a modest agreement between OCT and CLSM (correlation index of 0.55 (95 % CI: 0.33–0.71; *p* < 0.0001; Supplementary results; Fig. S1).

### 3.2 Effect of enzymatic treatment during *in situ* biofilm growth

**Fig. 2:**
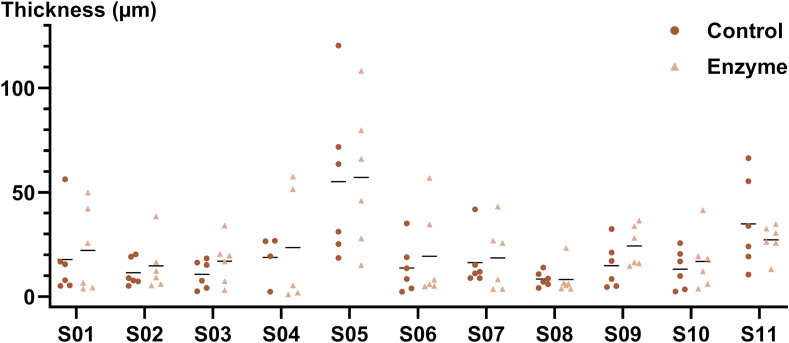
**Effect of enzymatic treatment during biofilm formation.** Enzyme or control treatment was performed three times per day for 30 min during *in situ* biofilm growth (48 h). Biofilm thickness was measured by OCT. No statistically significant difference in biofilm thickness was observed between groups (*p* = 0.15, gamma regression with log link function). Data from 129 biofilms (enzyme: *n* = 65; control: *n* = 64) from 11 participants (S01–S11) are shown (outliers: *n* = 3). Horizontal lines = means.

Enzymatic treatment during biofilm growth had no significant effect on biofilm formation, as determined by OCT. No difference in the average thickness was detected between enzyme treated (22.6 μm, 95 % CI: 16.1–31.6 μm) and control treated biofilms (19.5 μm, 95 % CI: 12.9–29.4 μm), with a ratio of means (enzyme/control) of 1.16 (95 % CI: 0.90–1.49; *p* = 0.15; Fig. 2).

### 3.3 Effect of enzymatic treatment on microbial biofilm composition

**Fig. 3:**
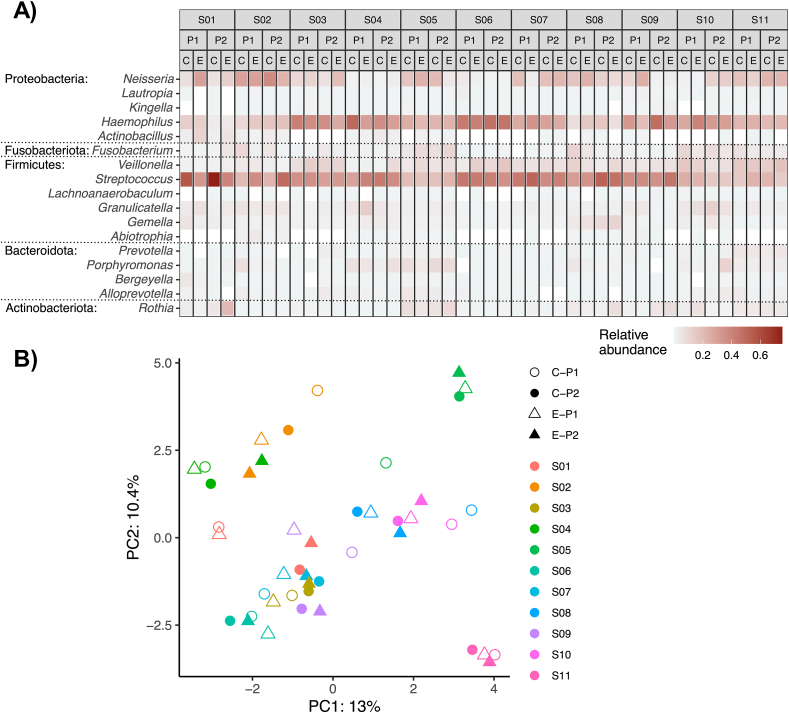
**The effect of enzymatic treatment on microbial biofilm composition. A)** The heatmap shows the most abundant genera (mean abundance cutoff = 1 %) in each analyzed biofilm, sorted by subject (S01–S11), period (P1 and P2) and intervention (E = enzyme-treated biofilm; C = control-treated biofilm). Biofilms were dominated by typical genera for young dental biofilms, with no systematic differences between intervention groups. **B)** Principal component analysis showed no clustering according to intervention group.

Biofilms in both intervention groups harbored typical microbiota for young dental biofilms (Supplementary File 2). The most abundant genera (Fig. 3A) were *Streptococcus* (34.1 % ± 12.9 % SD), *Haemophilus* (26.5 % ± 12.1 % SD), *Neisseria* (12.8 % ± 10.0 % SD), *Veillonella* (7.4 % ± 4.8 % SD) and *Fusobacterium* (3.1 % ± 3.4 % SD). The differential abundance analysis of paired enzyme and control treated biofilms collected from the same period did not identify any significantly different ASVs between the two groups (cutoff = 1 %). For some participants, marked compositional shifts were observed between the treatments, *e.g.* for S01, *Streptococcus* spp. were more abundant and *Neisseria* spp. less abundant in the control biofilms compared to enzyme-treated biofilms, while the opposite was observed for S02 in both intervention periods. Overall, biofilms from the same individual showed more similarity than biofilms from the same treatment group, as evidenced by principal component analysis (Fig. 3B).

### 3.4 Effect of enzymatic treatment on biofilm pH

**Fig. 4:**
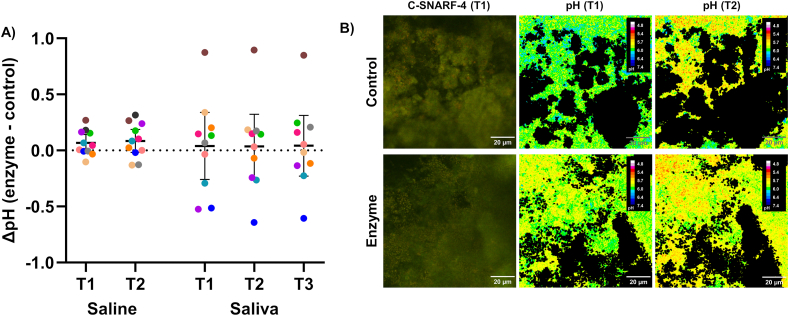
**The effect of enzymatic treatment on biofilm** pH**. A)** Average differences in pH between enzyme- and control-treated biofilms from the same individual (ΔpH) were 0.07 (T1) and 0.08 (T2) for sterile saline, and 0.04 (T1), 0.04 (T2) and 0.04 (T3) for cleared saliva. Each colored dot shows ΔpH for one participant. Horizontal lines = means; error bars = 95 % confidence intervals. Data from 43 biofilms (enzyme: *n* = 22; control: *n* = 21) from 11 participants (S01–S11) are shown (missing data: *n* = 1). **B)** Representative C-SNARF-4 images (left panels) of enzyme- and control-treated biofilms collected from one subject (S11) in period 1. The middle and right panels show the corresponding color-coded images of the extracellular pH at T1 (Control: pH 5.98; Enzyme: pH 5.90) and T2 (Control: pH 5.84; Enzyme: pH 5.83), respectively. T1 = 5 min after exposure to sucrose; T2 = 20 min after exposure to sucrose; T3 = 35 min after exposure to sucrose. (For interpretation of the references to color in this figure legend, the reader is referred to the Web version of this article.)

Biofilm pH developments upon sucrose exposure were assessed in both sterile saline and cleared saliva. In general, pH drops were slightly more pronounced in saline than in saliva, and the variation was smaller (Table 1). In both media, average biofilm pH did not differ significantly between intervention groups (Table 1, Fig. 4A). Representative ratiometric images of the typical pH response in enzyme-treated and control biofilms are shown in Fig. 4B. Null hypothesis b) was accepted.

**Table 1:** Biofilm pH (mean; 95 % confidence intervals) upon sucrose challenge in control- and enzyme-treated biofilms. Average biofilm pH was similar in both intervention groups. T1 = 5 min after sucrose exposure; T2 = 20 min after sucrose exposure; T3 = 35 min after sucrose exposure.

	Time	Control	Enzyme	*p*
**Saline**	**T1**	5.89 (5.83–5.96)	5.96 (5.89–6.04)	0.086
	**T2**	5.81 (5.72–5.90)	5.90 (5.81–5.99)	0.083
**Saliva**	**T1**	6.12 (5.91–6.33)	6.13 (5.88–6.38)	0.872
	**T2**	5.98 (5.73–6.23)	5.99 (5.68–6.30)	0.978
	**T3**	5.92 (5.67–6.18)	5.94 (5.61–6.27)	0.810

## 4 Discussion

This study investigated the effect of combined treatment with the three matrix degrading enzymes mutanase, beta-glucanase and DNase on biofilm formation and removal, on the microbial composition and on pH in a highly standardized *in situ-*model of dental biofilm. The enzymes were selected based on internal tests at Novozymes A/S and assessed as generally recognized as safe (GRAS). Under the chosen conditions, enzymatic treatment had no significant effect on the investigated outcomes compared to control treatment, and none of the null hypotheses could be rejected.

These results contrast with *in vitro* data for the same enzyme combination that showed a significant effect on biofilm prevention and removal [19]. The employed salivary *in vitro* model, however, consisted almost exclusively of *Streptococcus* spp. and therefore likely had a less diverse biofilm matrix that was more easily degraded by enzymatic treatment.

Moreover, our results contrast with two clinical pilot trials that found a significant reduction in the amount of plaque after one or eight days of treatment with the three investigated enzymes [48,49]. Several methodological differences may explain these conflicting results. One the one hand, biofilm formation in both clinical studies was assessed visually using clinical indices to determine plaque-covered areas [50,51]. Thereby, the whole tooth area was assessed, whereas quantification in the present study was performed on a small area. Moreover, biofilms were grown on an artificial substrate, which is a limitation of our study. On the other hand, plaque assessment by clinical indices is subject to both inter- and intra-observer variation [52], and the quantification does not include a measure of biofilm thickness, which was the central outcome in the present study. In this study, enzymatic treatment during biofilm growth was performed outside the oral cavity and hence at room temperature, which may have affected the enzyme activity of mutanase with an optimum between 40 °C and 56 °C [53]. In contrast, the pH of the employed enzyme solution (pH 6) was closer to the optimal range (pH 4.5 to 5.5) for mutanase activity compared to the pH in the oral cavity [53]. Moreover, the treatment duration of 30 min was longer than the treatment time in the clinical trials.

In line with the findings on biofilm formation and removal, we observed no systematic effect of enzymatic treatment on the microbial composition of the biofilms. Certain individuals showed treatment-induced bacterial shifts that were consistent across both intervention periods, but overall, the biofilm composition was primarily influenced by the participant and not by the treatment group. These results are in agreement with the above mentioned pilot trials that did not report any significant differences in the global microbial composition between intervention groups [48,49].

In addition to the microbial composition, we quantified the biofilm pH response to a sucrose challenge, to determine if potential effects of enzymatic treatment on biofilm formation or composition would result in reduced biofilm acidogenicity. Biofilm pH was not significantly different in the enzyme- and control-treated biofilms, which confirms that enzymatic treatment, under the chosen conditions, was not able to attenuate biofilm virulence in relation to caries development.

Biofilm formation in this study was assessed by CLSM and OCT, with a moderate correlation between both methods. While CLSM is routinely used for biofilm quantification, OCT has, as of today, only been employed in a few studies [26,54]. It allows for a rapid and label-free quantification of both fresh and fixed biofilms with a high penetration depth and a large sampling area. OCT also enables repeated measurements of the same biofilm before and after an intervention, but it has a lower spatial resolution than CLSM and it cannot distinguish between different biological components, such as epithelial cells, microbial cells and matrix polymers [55]. The fact that the quantification of treatment effects by OCT and CLSM yielded similar results suggests that both methods are suitable to quantify biofilm volumes.

Treatment effects of matrix-degrading enzymes are not as easily assessed as, *e.g.*, the effects of antibacterial agents. Møllebjerg et al. have shown that enzymatic treatment of biofilms can initially lead to swelling of the biofilm as the matrix composition changes. Hence biofilms appeared thicker, but were structurally weaker as the enzyme-treated biofilms could be removed more easily when subsequently applying mechanical stress [26]. Biofilm control by matrix-degrading enzymes therefore depends on the amount of shear applied to the biofilm, and the washing procedure in this *in situ* trial may not have been harsh enough to remove destabilized parts of the biofilms. The carriers used for biofilm collection were mounted on additively manufactured intraoral splints with a standardized geometry that improves reproducibility of replicate samples collected from the same participant [25]. However, the carriers were mounted in a recessed position and thereby protected from contact with the mucosa and hence, to some extent, from shear stress. During the washing step that was performed as part of both enzyme and control treatment, approximately half of the biofilm was removed in both intervention groups. It is conceivable that the remaining biofilm was sturdier and that the washing procedure therefore masked a potential effect of enzymatic treatment. Alternatively, the enzymes may have failed to fully penetrate the biofilms [12], or else have been prevented from degrading their target, *e.g.* by DNA-binding proteins such as DNABII that has been shown to stabilize eDNA into an enzyme-resistant form [56,57].

Within the limits of this split-mouth trial, it was not possible to include longer treatment times or a third intervention group as a positive control to prove that significant biofilm removal can be achieved. However, a recent study that investigated the effect of different multi-enzyme combinations on biofilm removal in a similar *in situ* model showed strong reductions for combinations of up to six different enzymes [15]. This suggests that a diverse cocktail of enzymes with different specificities may provide more efficient removal of *in situ-*grown biofilms.

In conclusion, treatment with a combination of mutanase, beta-glucanase and DNase did not significantly affect biofilm formation in the employed *in situ* model. Randomized controlled trials are needed to validate the promising results from clinical pilot trials on this enzyme combination. In addition, studies that map the components of the dental biofilm matrix to identify potential enzyme targets and their interactions, and studies that apply a broad range of enzymes may help to explore their full potential as a non-biocidal approach to dental biofilm control.

## CRediT authorship contribution statement

**Pernille Dukanovic Rikvold:** Conceptualization, Methodology, Investigation, Data curation, Visualization, Writing – original draft, Writing – review & editing. **Andreas Møllebjerg:** Methodology, investigation, data curation, software, visualization, Writing – review and editing. **Eero Juhani Raittio:** Formal analysis, Writing – review and editing. **Signe Maria Nielsen:** Methodology, Investigation, Writing – review and editing. **Karina Kambourakis Johnsen:** Investigation, Writing – review and editing. **Marie Braad Lund:** Investigation, Formal analysis, Visualization, Writing – review and editing. **Mette Rose Jørgensen:** Conceptualization, Methodology, Supervision, Resources, Writing – review and editing. **Rikke Louise Meyer:** Conceptualization, Methodology, Supervision, Resources, Writing – review and editing. **Sebastian Schlafer:** Conceptualization, Methodology, Investigation, Supervision, Resources, Writing – original draft, Writing – review & editing. All authors gave their final approval and agree to be accountable for all aspects of the work.

## Funding sources

This work was supported by Novozymes A/S and by the Innovation Fund Denmark (9065-00244B). The funders provided financial and administrative support, as well as the tested enzymes, but had no influence on data collection and on the decision to publish. All analyses were performed by Aarhus University. Mette R. Jørgensen provided supervision.

## Declaration of competing interest

The authors declare the following financial interests/personal relationships which may be considered as potential competing interests: Pernille Dukanovic Rikvold reports financial support was provided by Novozymes (Pernille Dukanovic Rikvold and Mette Rose Jørgensen were employed at Novozymes A/S when the study was performed). Pernille Dukanovic Rikvold reports financial support was provided by Innovation Fund Denmark. Patent #WO 2023/110,900 has been issued by Novozymes A/S. The other authors declare that they have no known competing financial interests or personal relationships that could have appeared to influence the work reported in this paper.

## Figures and Tables

**Table:** 

ASV	Amplicon sequence variant
CLSM	Confocal laser scanning microscopy
eDNA	Extracellular DNA
OCT	Optical coherence tomography
PBS	Phosphate buffered saline
PCB	Phosphate-citrate buffer

## Data Availability

The authors do not have permission to share data.
