# Supplementary material for: The effect of multiple-enzyme treatment on in situ oral biofilm formation in healthy participants
Source: Biofilm. 2025 Jun 21;10:100298. doi: 10.1016/j.bioflm.2025.100298 (PMC12266543; doi:10.1016/j.bioflm.2025.100298)
Supplement: Multimedia component 1 [file mmc1.docx]

**Title:** The effect of multiple-enzyme treatment on *in situ* oral biofilm formation in healthy participants

**Supplementary results**

*Comparison of optical coherence tomography (OCT) and confocal laser scanning microscopy (CLSM) for biofilm quantification*

Biofilm volumes quantified by OCT and CLSM showed a correlation index of 0.55 (Figure S1A). A flat relationship was found for small CLSM volumes (< 0.3 million μm^3^), which may be explained by lower resolution obtained with OCT compared to CLSM ([Wagner and Horn 2017](#_ENREF_3)). For CLSM values above 0.5 million μm^3^, a large spread was observed, which may, in part, be ascribed to the greater penetration depth of OCT than what can be achieved with CLSM ([Jonkman et al. 2020](#_ENREF_1); [Vroom et al. 1999](#_ENREF_2)). Figure S1B shows corresponding biofilm images acquired with OCT (blue panel) and CLSM (red panel).

**Supplementary figure** *
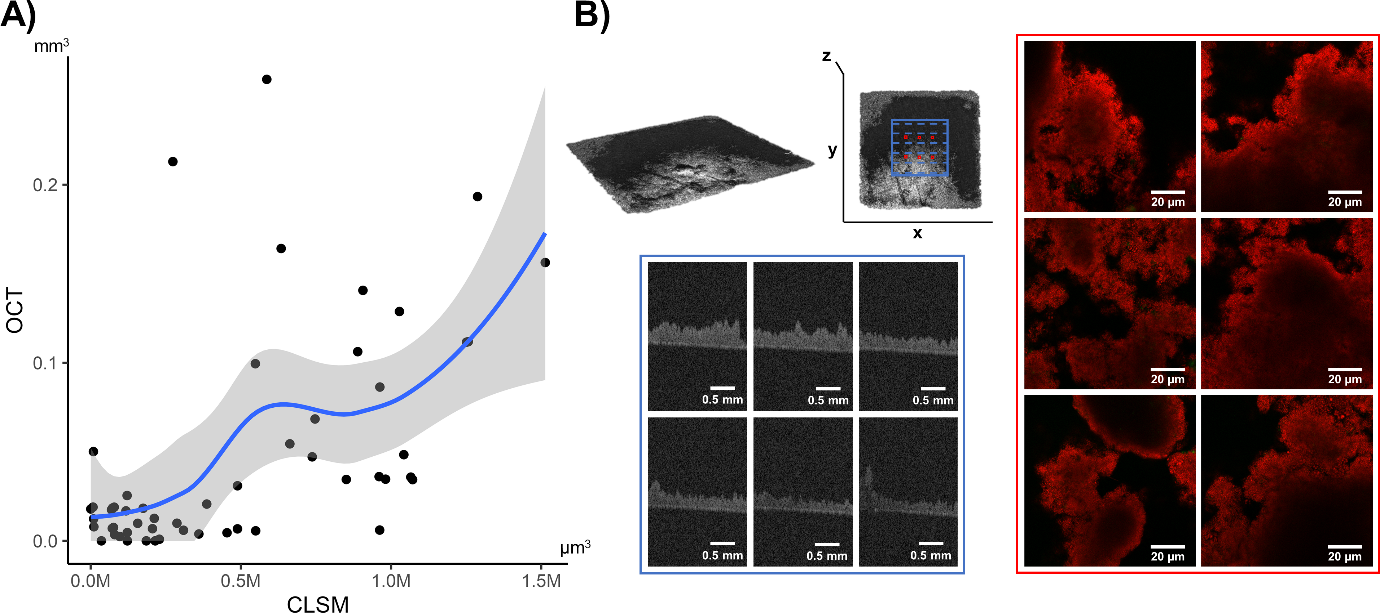
*

**Figure S1: The correlation between biovolumes quantified by optical coherence tomography (OCT) and confocal laser scanning microscopy (CLSM). A)** Correlation between both quantification methods (blue line) was moderate (0.55), with a flat relationship for CLSM values below 0.3 million μm^3^; for values above 0.5 million μm^3^, a large spread was observed. Dots represent individual biofilms (*n* = 54). Grey area = 95% confidence intervals. **B)** Top left images show a 3D rendering of an oral biofilm generated from OCT images viewed from two different angels. 500 vertical sections were acquired in the central 2 mm^2^ of the biofilm (blue square), and the average heights quantified. The bottom left images show six vertical sections from the biofilm above (dashed blue lines). For CLSM, six predefined field of views (red squares in the upper right image) were imaged by CLSM to determine the microbial biovolume (red image panel).

**Supplementary table**

**Table S1:** Biofilm biovolumes (mean; 95% confidence intervals) in control- and enzyme-treated biofilms, determined by confocal laser scanning microscopy. No statistical difference in biovolumes was observed between the two groups.

| **Treatment** | **Mean biovolume (µm^3^)** | **95% CI, lower (µm^3^)** | **95% CI, upper (µm^3^)** |
| --- | --- | --- | --- |
| **Control** | 98244 | 53056 | 181920 |
| **Enzyme** | 106999 | 54344 | 210672 |

**References:**

Jonkman J, Brown CM, Wright GD, Anderson KI, North AJ. 2020. Tutorial: Guidance for quantitative confocal microscopy. Nature Protocols. 15(5):1585-1611.

Vroom JM, De Grauw KJ, Gerritsen HC, Bradshaw DJ, Marsh PD, Watson GK, Birmingham JJ, Allison C. 1999. Depth penetration and detection of ph gradients in biofilms by two-photon excitation microscopy. Applied and Environmental Microbiology. 65(8):3502-3511.

Wagner M, Horn H. 2017. Optical coherence tomography in biofilm research: A comprehensive review. Biotechnology and Bioengineering. 114(7):1386-1402.
