# Supplementary material for: The effect of multiple-enzyme treatment on in situ oral biofilm formation in healthy participants
Source: Biofilm. 2025 Jun 21;10:100298. doi: 10.1016/j.bioflm.2025.100298 (PMC12266543; doi:10.1016/j.bioflm.2025.100298)
Supplement: Multimedia component 2 [file mmc2.pdf]

In [7]:

```python
# Before quantifying the biovolume, we need to tell the program what is a signal and what is background.
# We do this by segmentation according to a certain threshold. Hence any pixel with an intensity below that threshold
# will not be included in the signal, but as background noise. We can set the threshold based on a intensity histogram

import numpy as np # These functions need to be imported for the program to run
import skimage
from PIL import Image
from numpy import asarray
from matplotlib import pyplot as plt
from skimage import morphology, filters
from skimage.measure import label, regionprops
from skimage.color import label2rgb

#--------Must be set---------------------------------------------------------------------
Threshold = 50 #set lower threshold based on histogram

top=50 # How much to crop from the top and bottom of the images. This can be determined before
bottom=0
#--------Can be changed------------------------------------------------------------------
path='Slice Folder' # The path of the image
testimage='4' # We import a test image and slice to determine a proper threshold
testslice='0148'
filetype= '.png'

FilterFactor = 5
#---------------------------------------------------------------------------------------
im=Image.open(path+'\\'+testimage+'_'+testslice+filetype).convert('L')
w,h=im.size
imc=im.crop((0, top, w, h-bottom))
im1 = np.array(imc)# Load the image as np array in grayscale
img = filters.median(im1, morphology.disk(FilterFactor))

plt.hist(img.flatten(),100,range=[0.000001,max(img.flatten())]) # Plot the pixel intensities in a histogram, 50 bins, plot excluding 0
plt.plot([Threshold, Threshold], [0, len(img.flatten())/100], 'r-') # Indicates threshold value on histogram (red line)
plt.title('Threshold')
plt.show()

Signal = (Threshold < img) # Define signal pixels
Background = (Threshold > img) # Define background pixels


Biofilm = label((Signal * 255).astype(np.uint8)) # label part of image defined as biofilm
Labeled_Biofilm = skimage.color.label2rgb(Biofilm, image=img, bg_label=0, colors=['red']) # Create overlay with original image

fig, ax = plt.subplots(1, 3, figsize=(15,5)) # figsize sets size in inches
ax[0].imshow(im, cmap='gray')
ax[0].set_title('Original')
ax[1].imshow(img, cmap='gray') # Show threshold segmented images. Change threshold until satisfactory
ax[1].set_title('Cropped & Filtered')
ax[2].imshow(Labeled_Biofilm, cmap='gray') # Show threshold segmented images. Change threshold until satisfactory
ax[2].set_title('Identified Biofilm')
plt.show()


# When the threshold is appropriate, proceed to the next step
```

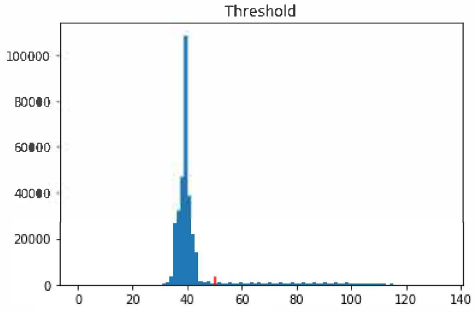

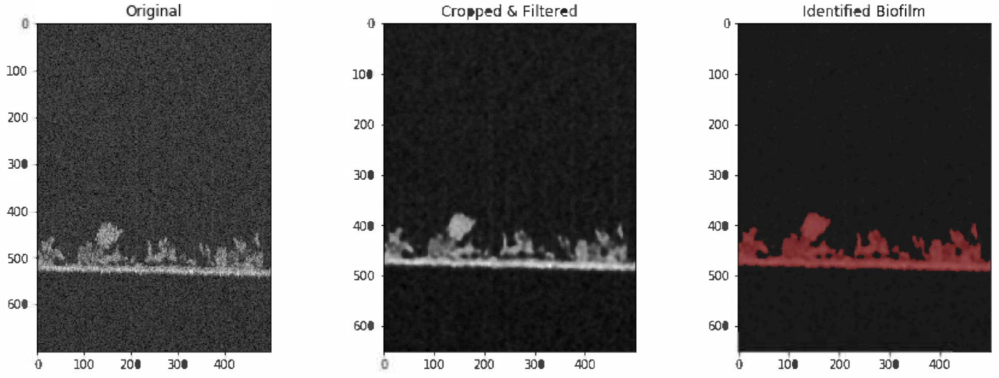

In [1]:

```
# Quantifying 3D height in several volume scans
import time
import xlsxwriter
from time import process_time
import pandas as pd
import os
import numpy as np
import seaborn as sns
from numpy import asarray
from PIL import Image
from matplotlib import pyplot as plt
from skimage import morphology, filters


#--------Must be set-----------------------------------------------------------------------------------------------------
VolumeScans=66 # number of volume scans

Threshold = 50 #set threshold based on histogram
TopCrop, BottomCrop=50, 0 # How much to crop from the top and bottom of the images. This can be determined before

ShutdownPC = "n"
SaveHeatmap='y' #Choose whether to save the graphs as image files (y/n)

#--------Can be changed--------------------------------------------------------------------------------------------------
Path='Slice Folder' # Here we define the name of the subfolder that our images are located in.
FileType= '.png'

Delay = 0 # Delay in minutes before image analysis

FilterFactor = 5 # Defines how coarse the filtering is

PlateRows = 6 # Write how many rows are the plate (for heatmap)
#-----------------------------------------------------------------------------------------------------------------------
if Delay>0:
    print('Analyzing images in: '+str(Delay)+' minutes')
tic = process_time()

# First we define the image dimensions using the txt file in the folder
dimensionFile = open("Dimensions.txt", "r")
dimensionlst = (dimensionFile.readlines())
dimensionstr1 = "".join(dimensionlst).replace('\n','')
dimensionstr2 = dimensionstr1.split(' , ')
dimensions = np.array(dimensionstr2).astype(np.float)
dimensionFile.close()

SlicesPerVolume=int(dimensions[3]) # number of slices per volume scan
xVox, yVox, zVox= [dimensions[2], dimensions[1], dimensions[0]] # The voxel dimensions in the x, y and z direction (mm)

# The following list is used to generate a string of numbers that is used later to refer to the sample names
VolumeList=[]
for i in range(VolumeScans):
    nr=i+1
    VolumeList.append(str(nr))

#----Now we create two lists that are used in the image analysis-----------------------------------------------------------------------------
SliceList=[]
for i in range(SlicesPerVolume): # The list allows us to import up to 9999 images per sample
    nr=i+1
    if i < 9:
        SliceList.append('000'+str(nr))
    elif i < 99:
        SliceList.append('00'+str(nr))
    elif i < 999:
        SliceList.append('0'+str(nr))
    else:
        SliceList.append(str(nr))

time.sleep(Delay*60)

# Now we create matrices in which to store our image data.
RandomVolume, RandomSlice='1','0001' # We import a random image and slice to extract the slice dimension for the matrix
imi=np.array(Image.open(Path+'\\'+RandomVolume+'_'+RandomSlice+FileType).convert('L'))
SliceHeight, SliceWidth=imi.shape
matrixk = np.zeros(shape=(SliceWidth, SlicesPerVolume), dtype=float) # Here we create the matrix to pick up data from one volume
HeightData=pd.DataFrame()
print("Image treatment beginning")
# Now we actually extract our data from the images. The program does this by applying the same mask to the image as above
# before extracting the height of the sample for every xy position in a volume scan, and storing them in the matrix.
# Every row of the matrix contains a list of all the height positions for one volume scan
for k in range(VolumeScans):
    for i in range(SlicesPerVolume):
        FileName='\\'+VolumeList[k]+'_'+SliceList[i]
        img=np.array(Image.open(Path+FileName+FileType).convert('L'))
        h,w=img.shape
        imi2 = Image.fromarray(img)
        imi3 = imi2.crop((0, TopCrop, w, h-BottomCrop))
        img = np.asarray(imi3)
        hc,wc=img.shape
        im = filters.median(img, morphology.disk(FilterFactor))
        signal = (Threshold < im)
        matrixk[:,i]=np.sum(signal*zVox,axis=0)
    VolumeName=str(k+1)
    HeightData[VolumeName]=matrixk.flatten().tolist()
```

```
    print("Volume "+str(k+1)+" processed")

matrixk=[]
HeightData=HeightData.replace(0, np.NaN)
HeightStats=pd.DataFrame(columns=HeightData.columns)
RoughStats=HeightData.copy(deep=True)
HeightStats.loc['Mean Thickness (\u03BCm)']=pd.Series(HeightData.mean())
HeightStats.loc['Std. Dev.']=pd.Series(HeightData.std())
HeightStats.loc['Min']=pd.Series(HeightData.min())
HeightStats.loc['Median']=pd.Series(HeightData.median())
HeightStats.loc['Max']=pd.Series(HeightData.max())
for i in range(1, VolumeScans+1):
    MeanHeight=HeightStats[str(i)].loc['Mean Thickness (\u03BCm)']
    RoughStats[str(i)]=abs(RoughStats[str(i)]-MeanHeight)/MeanHeight
HeightStats.loc['Roughness Coefficient']=pd.Series(RoughStats.mean())
HeightStats.loc['Volume (\u03BCm\u00b3)']=pd.Series(HeightData.sum()*xVox*yVox*1e-9)
HeightData=HeightData.replace(np.NaN, 0)

SortingDict={'Mean Thickness (\u03BCm)':0, 'Std. Dev.':1, 'Min':2, 'Median':3, 'Max':4, 'Roughness Coefficient':5, 'Volume (\u03BCm\u00b3)':6}
HeightStats.reindex(SortingDict)
Height=pd.concat([HeightStats,HeightData])
#HeightStats.to_excel('Data Folder\\Height.xlsx')

# #-----------------------------------------------------------------------------------------------------------------------------
# # Now we plot a heatmap of the volume distribution over the plate
HeatmapArray=HeightStats.loc['Volume (\u03BCm\u00b3)'].to_numpy()
Heatmap=np.reshape(HeatmapArray, (PlateRows, (VolumeScans//PlateRows)), order='F')

x_labels=np.arange(1, (VolumeScans//PlateRows)+1)
y_labels=np.arange(1, PlateRows+1)
Heatmap_df=pd.DataFrame(Heatmap)
ExcelWriter = pd.ExcelWriter('OCT Data.xlsx', engine='xlsxwriter')
try:
    Height.to_excel(ExcelWriter, sheet_name='Raw Data')
except:
    HeightStats.to_excel(ExcelWriter, sheet_name='Raw Data')
    del HeightData
    del Height
    print('Dataset too large: only statistical data stored')
Heatmap_df.to_excel(ExcelWriter, sheet_name='Volume')
worksheet1 = ExcelWriter.sheets['Raw Data']
worksheet1.set_column(0, 0, 22)
worksheet2 = ExcelWriter.sheets['Volume']
worksheet2.write_row('B1', x_labels)
worksheet2.write_column('A2', y_labels)
worksheet2.conditional_format('B2:K7', {'type': '3_color_scale', 'min_color': 'green', 'max_color': 'red'})
ExcelWriter.save()

fig = plt.figure(figsize=(8,3)) # Set plot size
ax = sns.heatmap(Heatmap, xticklabels=x_labels, yticklabels=y_labels, linewidth=0.5, cmap='RdYlGn_r')
plt.title('Volume heatmap', fontsize=20)
if SaveHeatmap == 'y':
    plt.savefig('Data Folder\\Volume Heatmap.png', dpi=600, bbox_inches='tight')
plt.show()
# #-----------------------------------------------------------------------------------------------------------------------------

toc = process_time()
hours =int((toc-tic)/3600)
minutes = int(((toc-tic)-(hours*3600))/60)
seconds = int((toc-tic)-(minutes*60))
print(" Program finished. Processing time: ", hours, " hr, ", minutes, " min, ", seconds, " sec")

if ShutdownPC == 'y': # shuts down the PC 1 minute after data treatment if 'y'
    os.system("shutdown /s /t 60")
```
